# Computational investigation of the control of the thermodynamics and microkinetics of the reductive amination reaction by solvent coordination and a co-catalyst†

**DOI:** 10.1039/c8ra08135b

**Published:** 2018-10-30

**Authors:** Esra Boz, Nurcan Ş. Tüzün, Matthias Stein

**Affiliations:** Max Planck Institute for Dynamics of Complex Technical Systems, Molecular Simulations and Design Group Magdeburg Germany matthias.stein@mpi-magdeburg.mpg.de; Istanbul Technical University, Department of Chemistry Istanbul Turkey

## Abstract

Amines are among the most important and frequently used chemical compounds due to their biological activity and a wide range of applications in industry. Reductive amination reactions are an efficient and facile route to synthesize long chain amines from sustainable sources by using a different available aldehydes and ketones, and a large variety of amines including primary, secondary and tertiary forms. The pathway of the reaction process is critically dependent on reaction parameters such as the pH of the reaction medium, choice of solvent (explicitly coordinating solvent) and process conditions. These parameters are affecting the reaction performance and the selectivity but are still not fully rationalized. Here, we investigate the microkinetics and thermodynamics of the individual steps of the reductive amination reaction by exploring the systems' parameters. Explicit water coordination to the aldehyde leads to a stepwise rather than concerted nucleophilic addition with a lower activation barrier by 6–10 kcal mol^−1^. At low pH, the pathway is changed by a direct protonation of the amine substrate. This protonation does not strongly affect the kinetics of the reaction, but the thermodynamic equilibria. The presence of an acid as a co-catalyst leads to the formation of an iminium intermediate and this drives the reaction forward. Thus, the presence of an acid as a co-catalyst clearly renders this pathway the thermodynamically preferred one. Consequently, altering the reaction parameters does not only influence the reaction kinetics, but also the thermodynamic profile of the pathways in all cases. Further understanding of the reaction dynamics is essential to develop a microkinetic model of the reaction to then control and engineer the process in order to rationally design routes to tailor-made products.

## Abstract 2

Green production of tertiary amine from sustainable sources can be controlled by appropriate choice of reaction parameters identified by computational means.

## Graphical abstract

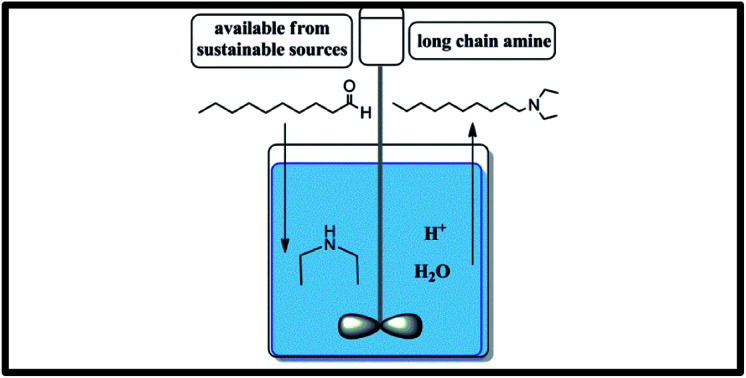

## 1. Introduction

**Scheme 1:**
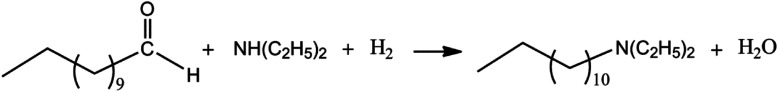
Production of long chain diethylamines from aldehydes *via* reductive amination.

A green chemistry approach to the production of fine chemicals and the utilization of natural products from sustainable sources is becoming more significant in industrial applications. Amines represent an important class of high-quality chemicals which are used as intermediates in a range of applications including pharmaceuticals, agricultural chemicals, rubber chemicals, water treatment chemicals, and also as solvents.^1^ The transformation of industrially available fatty acids from sustainable sources into long chain amines *via* an environmentally friendly and sustainable reaction has recently gained significant importance (Scheme 1).^2^ Indeed, the reaction of reductive amination has been recognized to be one of the most relevant challenges for process design for the pharmaceutical industry.^3^ The sequential or tandem coupling of industrial chemical processes^4^ gives an elegant access to these classes of compounds, for example by coupling a hydroformylation reaction which is followed by an amination of the obtained aldehyde and finally a hydrogenation of the enamine to produce saturated, long chain primary, secondary or tertiary amines.^5^ Starting from *n*-undecene, first *n*-dodecanal (lauryl aldehyde, a fragrance) is being produced which is then converted into *N*,*N*-diethyltridecane-1-amine (C_15_H_33_N, an anti-infective; Scheme 1).

Such a facile and efficient route for a hydroaminomethylation (HAM) process is of great interest to the chemical and pharmaceutical industry.^6,7^

**Scheme 2:**
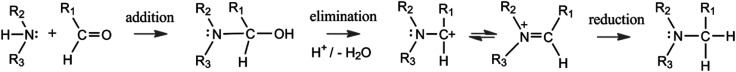
Proposed mechanism for the reductive amination of aldehydes.

The reductive amination initiates with the addition of an amine to the carbonyl group of the aldehyde/ketone and a hemiaminal as intermediate is formed (Scheme 2). The subsequent condensation reaction results in an imine or an iminium ion depending on the pH of the reaction medium. The equilibrium between aldehyde/ketone and imine can be controlled by continuous removal of the released water. At the final step, the desired amine is obtained by a reduction of the C=N bond by using either an organic hydride donor or a transition metal catalyst. However, reduction of the C=N bond in an efficient, industrially applicable and non-toxic way is still an area of intense research.

Due to their great importance, there are numerous synthetic approaches to form amines. These methods are generally classified as ‘direct’ and ‘indirect’ according to the instance of addition of the reducing agent.^8^ The reaction is described as ‘direct’, when aldehyde, amine and reducing agent are reacting in a one pot process.^5^ The drawback of the direct reduction process is the competition between reduction of the C=O carbonyl group of the aldehyde and the C=N unsaturated bonds of imine which leads to a number of side products. Thus, the appropriate choice of the reducing agent becomes more critical. In order to obtain an amine as a final product, the reducing agent must react selectively with the imine (or enamine, when secondary amines are used) or iminium ion rather than the carbonyl group of the aldehyde. Usually, direct reductive amination is preferred over an ‘indirect’ process, because it is more conveniently handled and a more efficient process, especially in large scale applications.^4^ The use of borohydrides, for example sodium triacetoxyborohydride,^8–10^ is one example of a simple and cheap choice of reducing agents because of its different selectivity at different pH, its stability in acidic conditions (pH = 3) and its solubility in polar solvents. However, the toxicity of the reducing agent and its low conversion rate in case of unreactive ketones hamper the large scale application.^10^ Recently, a silane-based reductant was reported for the direct reductive amination to give only one representative of the organosilane family.^11^ This reducing reagent is not only cheap and metal free to classify it as a green process; but it also shows a high selectivity to the C=N bond which prevents the formation of undesired side products.

Heterogeneous catalytic reductive amination with suitable transition metals is an efficient and economical way and used frequently on industrial scale and this procedure is successful with noble metal complexes such as Rh, Ru, and Pd/C systems.^12,13^ Homogeneous transition metal catalysts such as Rh(i), Ru(ii) and Ir(i–iii)^14–16^ are also successful tools for the asymmetric reduction of imines/enamines when in complex with different ligands.^17^ These reducing agents are particularly used for an enantioselective synthesis of amines.

Most of the reduction methods are established for the synthesis of primary and secondary amines. However, production of a tertiary amine is more challenging due to the steric demand of two nitrogen-bound organic rests (R_2_ and R_3_ in Scheme 2) and the strained formation of the imine/enamine intermediates. Several metal-based catalyst systems (such as Co_3_O_4_/H_2_ and SnCl_2_/reducing agent) are mentioned in the literature as suitable candidates for this process.^11,18,19^ There are also several successful experimental and computational reports on the functionalization of several amines *via* intra- and intermolecular cyclization reactions in a redox neutral way.^20–22^ In addition, they give some evidence for the formation of ylide intermediates which suggests that the reaction can also proceed *via* an alternative route and would not necessarily involve the formation of iminium ions. A recent and efficient method is the photocatalytic reduction of iminium ions to produce amines and further functionalize them into diverse drug-like compounds.^23^ Computational approaches can reveal mechanistic pathways and alternatives.^24^ Previous computational studies have focused on the direct reductive amination reaction in terms of transition metal catalysed hydrogenation or the stereoselectivity.^25–32^ The mechanism of enamine formation was investigated using DFT calculations and different functionals.^33^

Processes in chemical industry are undergoing a large change towards the use of feedstock from renewable sources. In a recent study, a catalytic hydroaminomethylation process from sustainable substrate has been introduced to give industrially important surfactant products.^6^

Here, we present a computational study on the thermodynamics and full mechanism of the reductive amination reaction in an organic solvent in the absence and presence of the co-catalyst acid using a combination of an explicit cluster model of solvation and an implicit solvent model to account for long range effects. Information about the thermodynamics, the non-ideality of reaction media and the rate-determining step are required for the design of a reaction network model of the reductive amination of sustainable long-chain aldehydes such as tridecanal which can elegantly be generated from a Rh-based hydroformylation reaction of 1-dodecene in a thermomorphic multicomponent solvent system (TMS)^34–37^ or directly in a hydroaminomethylation reaction in a TMS.^38^

We here investigate in detail the reductive amination mechanism of propanal by diethylamine to yield *N*,*N*-diethylpropane-1-amine under different reaction conditions:

(a) The reaction in neutral media with an implicit solvent model.

(b) The reaction in the presence of explicitly considered solvent molecules (here water) to form a hydrogen bond network between electrophilic and nucleophilic sites plus an implicit solvation model.

(c) The effect of the co-catalyst acid on the reaction profile.

## 2. Methods

In the scope of this work, a representative reaction between a long chain aldehyde and a secondary amine has been studied in terms of neutral and co-catalyst assisted reaction conditions. In order to simplify the system and reduce the conformational space, propanal was chosen to replace dodecanal (C_12_H_24_O). The calculation of the Gibbs free energy of the reaction shows, however, that the choice of model does not affect the thermodynamics and thus the reaction equilibrium.

The reductive amination process is investigated using *ab initio* Møller–Plesset perturbation theory (MP). The consideration of electron correlation is crucial to accurately describe the electronic structure of such a system and has to be taken into account which is not fully considered in density functional theory (DFT). The barrier heights of an enamine formation calculated at DFT and the second order Møller–Plesset perturbation theory (MP2) levels were shown to differ by up to 4 kcal mol^−1^ which is larger than ‘chemical accuracy’. In addition, the computed energy barriers in an implicit solvent were found to be lower by 14 kcal mol^−1^ than in gas phase.^33^ This is also emphasizing the importance of an accurate consideration of solvent effects and the stabilizing effect of explicitly coordinating solvent molecules on the charge distribution and that solvent effects should be taken into account to refine the computed barriers.

All the intermediates and transition state structures were fully optimized by using MP2(full) with a 6-311+g (d,p) basis set^39^ in Gaussian 09.^40^ Optimized structures were verified by calculation of second derivatives to correspond to minima, whereas a single imaginary frequency characterize a transition state. In order to verify the transition state structures, intrinsic reaction coordinate (IRC) calculations were done along the reaction coordinate by following the gradient in both directions and were found to connect the transition states with the educts and products, respectively.^41,42^ All the energies reported through this work are Gibbs free energies at standard conditions of 298 K and 1 bar. Thermodynamic corrections were taken from MP2 frequency calculations at 298 K and added to the single point SMD MP2 energies. Full energies plus thermodynamic corrections of all species are given in the ESI.† Charge analysis on the optimized structures was performed using the Natural Bond Orbital (NBO) method.^43,44^ The bond distances are given in units of Å written in black and NBO charges in blue where appropriate.

Single point implicit solvent calculations were carried out at the optimized geometries with the same level of theory in order to estimate the effect of the solvent on the reaction profile. The solvent effects were included using the solvation model based on density (SMD) method for *N*,*N*-dimethylformamide (DMF) as a polar and decane as an apolar solvent to mimic the polarity effect (*ε* = 36.7 and 2, respectively).^45^ The choice of solvents correspond to that used in the experiments for the hydroformylation reaction of dodecene.^35,36^ In the main text of this manuscript, we only report Gibbs free energies in the presence of DMF and decane. Results of calculations in the gas phase can be found in the ESI† for all reaction pathways. Generally, polar and apolar solvents have a strong effect on the thermodynamics and kinetics of the reductive amination reaction. Since we are focusing on the control by choice of solvent, only the results in solution are given in the main text.

## 3. Results and discussion

### 3.1 Reaction thermodynamics

The thermodynamics of a reaction are critical parameters for simulations of chemical reaction networks^46^ and entire processes.^47^ Standard thermodynamic parameters such as reaction enthalpy and Gibbs free energy of the ideal system are often not available in the literature but can be obtained computationally and then later combined with other thermodynamic approaches to account for the non-ideality of complex reaction mixtures at process conditions.^35^ Solvent effects on the kinetics^48^ and thermodynamics^49^ of the hydroformylation reaction of 1-dodecene were already investigated experimentally and combined with the Perturbed Chain Statistical Associating Fluid Theory (PC-SAFT) to model the reaction of dodecene with syngas (CO/H_2_) in a solvent mixture of DMF/decane at 90 °C and 21 bar. Quantum chemical calculations of thermochemical data pose a challenge in terms of accuracy and standard DFT functionals were not suitable to obtain accurate data for the hydroformylation of long chain olefins but MP2 calculations gave reliable thermodynamic equilibrium constants *K*_f_.^49^

The reaction thermodynamics 
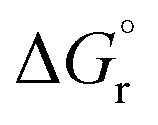
 of the reductive amination reaction of aldehydes of various carbon chain lengths and diethylamine were calculated in the gas phase and in an implicit solvent environment. The free energy of the reaction was −14.7 kcal mol^−1^ and only slightly affected by presence of a solvent to become −15.1 and −15.3 kcal mol^−1^ in either DMF and decane (see Table 1).

**Table 1:** The thermodynamics (
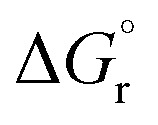
, kcal mol^−1^) and solvent effects of the reductive amination reaction of various aldehydes with diethylamine ((C_2_H_5_)_2_NH)

Aldehyde	Free energy of reaction ( 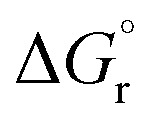 , kcal mol^−1^)		
	Gas phase	DMF	Decane
C_3_H_6_O	−14.7	−15.1	−15.3
C_5_H_10_O	−14.5	−15.4	−15.2
C_9_H_18_O	−14.5	−15.3	−15.1
C_10_H_20_O	−14.4	−15.3	−15.1
C_11_H_22_O	−14.3	−15.2	−15.0

Then, the effect of the chain length of the aldehyde on the thermodynamics of the reductive amination reaction was investigated. The free energy of the reaction is almost independent of the chain length and between −14.3 and −14.7 kcal mol^−1^ for all carbon chain lengths, and the effect of the solvent is only minor.

One can thus conclude that the thermodynamics of the reaction is independent from the chain length of the aldehyde and a truncated substrate model is appropriate. Thus, we investigated the mechanism of the reductive amination only for the short chain aldehyde, *e.g.* for the reaction of propanal and diethylamine.

### 3.2 Reaction mechanism

Reductive amination of an aldehyde starts with a nucleophilic addition of the amine to the carbonyl group of the aldehyde. The initial step of the reaction leads to the formation of a carbinolamine intermediate. In an intramolecular condensation reaction, an enamine or an iminium ion are formed depending on the chosen reaction conditions (in particular the pH of the reaction medium). This enamine formation is an equilibrium reaction and can be controlled by H_2_O addition or removal in a chemical process. At the final step, the product amine is generated by reduction of the enamine or iminium. Throughout the reaction process, there are several key structures and the reaction is proceeding *via* multiple transformations in order to form the tertiary amine as a final product. These chemical transformations are controlled by several factors like the pH of the reaction medium (see above), the polarity of the solvent, explicit formation of hydrogen bonds by coordinating polar reaction media, and activation of the intermediates by Lewis base/Brønsted acid addition. During an automated optimization the amine formation during reductive amination takes around 2 minutes with moderate to high yields (67–97%). The conversion rate is lowered by the steric amines and results in byproduct formation *via* simple reduction.^50^

#### 3.2.1 The reaction in neutral media

**Scheme 3:**
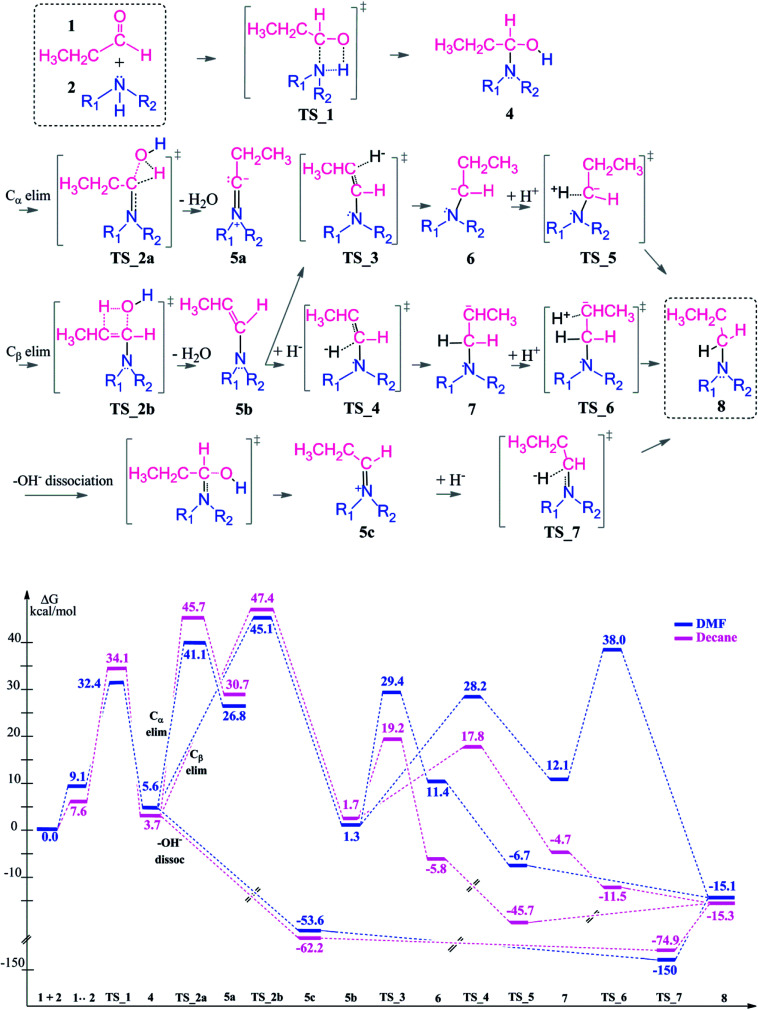
Reaction mechanism including transition states and intermediate structures (top) and the free energy profile for reductive amination reaction in neutral media (bottom), (R_1_ = R_2_ = –CH_2_CH_3_).

In neutral media, the aldehyde (1) and the amine (2) form a pre-complex before the nucleophilic addition of the lone pair of the nitrogen to the positively polarized carbon atom of the aldehyde occurs (Scheme 3) in a barrierless process. This pre-complex is an intermediate and 9.1–7.6 kcal mol^−1^ higher in energy than the substrates. Patil and Sunoj also report a barrierless nucleophilic attack from DFT mPW1PW91 calculation and an increase by 5 kcal mol^−1^ upon pre-complex formation.^33^ A hemiaminal intermediate (4) is formed *via* an intramolecular proton transfer from the amine to the carbonyl oxygen in a concerted fashion (TS_1) with an activation barrier of 33.3 kcal mol^−1^ (see ESI, Table S1†) in the gas phase and 32.4–34.1 kcal mol^−1^ in DMF and decane, respectively, which agree with the 38 kcal mol^−1^ in the gas phase (24 kcal mol^−1^ in THF).^33^ The polar solvent DMF only slightly stabilizes the transition state. A co-crystal structure of a hemiaminal and diethylamine complex gives an increase in bond length of C–OH from 1.410 to 1.430 Å which is in excellent agreement with the calculated 1.420 Å for our hemiaminal structure (4).^51^ Moreover, the position and the interactions of the hydrogen atoms in the co-crystal structure were suggested to be an indicator of a concerted mechanism which was also observed in the computations (TS_1).

The first coupling step is followed by an intramolecular condensation reaction and release of a water molecule, formed by the –OH group with a proton from the hemiaminal (4), see Scheme 3.

**Fig. 1:**
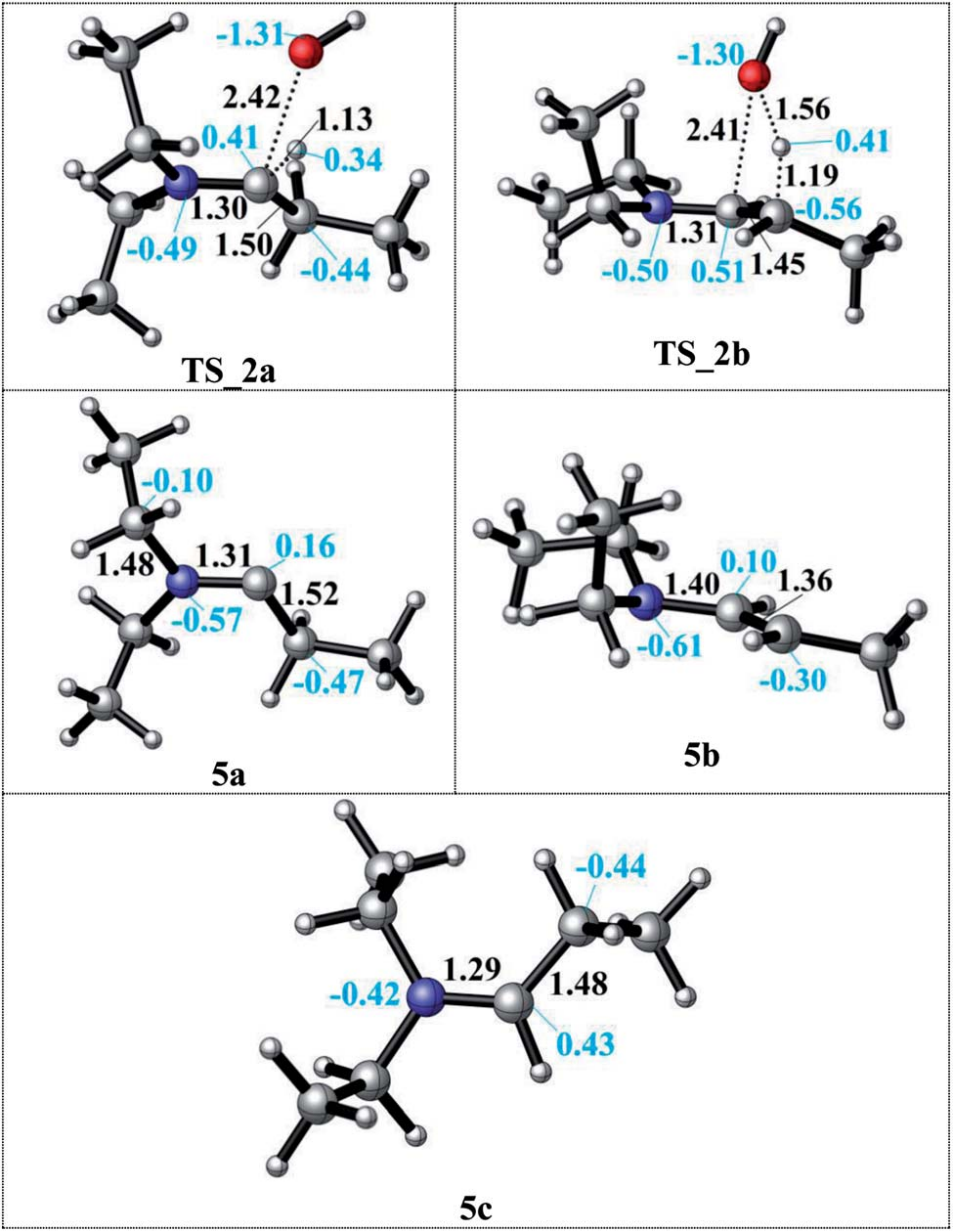
Details of the formation of the 5a and 5b intermediates after proton abstraction to release water *via*TS_2a (C_α_ position) and *via*TS_2b (C_β_ position) and the formation of the iminium ion 5c upon OH^−^ dissociation.

In the condensation step, a proton from either an α– or a β–carbon atom can be eliminated. Abstraction of the proton at α position *via*TS_2a leads to formation of the 5a intermediate whereas when the β-proton is eliminated *via*TS_2b and the enamine intermediate 5b is formed. The activation barriers for both routes are 48.9 kcal mol^−1^ in the gas phase. They are lowered by 5–10 kcal mol^−1^ in a solvent (but still larger than 40 kcal mol^−1^ in both DMF and decane solvents) with the α-pathway being slightly favoured. This is significantly lower than the 58–60 kcal mol^−1^ reported in the literature.^33^ The resulting intermediates 5a and 5b (enamine) are very different in stability (Scheme 3, bottom). The energy difference of 5a by 25–29 kcal mol^−1^ compared to 5b can be attributed to the larger charge separation in the structure and the re-hybridization character of the C_α_. Whereas in 5b, the newly formed double bond has a planar geometry with a *trans* orientation of the alkyl groups and a charge density that is evenly distributed along the structure. These are the factors which are stabilizing 5b with respect to 5a (see Fig. 1).

After examining the elimination from α and β positions, we are also considering the possibility of the dissociation of the hydroxyl anion without abstracting any proton (Scheme 3). This dissociation can occur by assistance of the solvent or the counter cations in the reaction medium. As a result of this dissociation, an iminium intermediate (5c) can be generated. This commonly accepted intermediate is formed in a highly exergonic fashion (around −54 to −63 kcal mol^−1^). The elongation of the C–OH bond takes place easily and without any barrier to release OH^−^ and results a stable iminium cation.

The final step in the reductive amination is the hydrogenation (reduction) of intermediates 5a, 5b and 5c to generate the desired tertiary amine. Besides the conventional organic boro hydride derivatives, there are several novel approaches for a mild and selective reduction and most of them consider a transition metal (mostly Ru and Rh) catalyzed hydrogenation.^52^ A kinetic study suggests that the rate of the reaction is independent of the imine concentration and organoborane catalyzed reduction is the rate determining step.^53^ In this work, we investigate a sequential approach for hydrogenation since that reflects also the procedure when an external hydride donor or an activated H_2_ molecule bound to a transition metal catalyst is used. We did not consider the hydride donor or the catalyst in molecular details since this is highly dependent on each experimental set-up and the choice of the reducing agent.

Since the enamine 5b is 25–29 kcal mol^−1^ lower in energy than 5a, only reduction of the enamine was investigated by addition of first the hydride (H^−^) and then the proton (H^+^) species. The hydride addition to the enamine 5b structure was investigated for both carbon atoms of the C=C double bond (C_β_*via*TS_3 to give 6 and C_α_*via*TS_4 to give 7). The C_α_ atom in 5b has a charge of 0.10, where C_β_ is negatively charged (Fig. 1). Thus, the attack of the H^−^ to the positively charged C_α_ carbon *via*TS_4 to generate structure 7 is found to be kinetically favourable with a difference in transition state barriers of 1.2–1.4 kcal mol^−1^ but thermodynamically less preferred by 0.7–1.1 kcal mol^−1^. Thus, we consider the presence of both intermediates 6 and 7 in the protonation step to give the final product 8. Intermediate 6 has a higher proton affinity by 44–47 kcal mol^−1^ than 7 and consequently the proton addition *via*TS_5 is thermodynamically preferred. TS_5 was found to be lower in energy than TS_6 in decane but upshifted by around 40 kcal mol^−1^ in DMF due to the solvent shielding of electrostatics. This destabilization leads to a barrier of around 26 kcal mol^−1^ for TS_6 in DMF which was exothermic in case of TS_5. The large destabilizing effects for the transition states TS_5 and TS_6 in polar media such as DMF indicate the need for a careful design of a suitable solvent to thermodynamically and kinetically enable the reaction.

The reduction of the intermediate 5c is quite distinct from the one of 5b. Due to the positive charge on the iminium ion, only hydride addition is sufficient to obtain the final product amine. Here, hydride addition occurs exergonically by −75 to −150 kcal mol^−1^. It is also noteworthy to mention that the polar solvent DMF stabilizes the charged transition state TS_7 and highly favors the hydride addition. As a result, reduction of 5c is preferred over that of 5b.

In conclusion, once the hemiaminal (4) is formed *via* the coupling reaction, transformation to the final product is thermodynamically favorable. This transformation will preferably take place *via* the iminium intermediate (5c) which is more stable than 5b and once again due to highly exergonic single step reduction to give the long chain amine.

#### 3.2.2 Explicit water-coordination

Water is the prime green solvent of a chemical reaction and was also shown to accelerate the reductive amination reaction itself.^14^ It is also observed that H_2_O addition has a positive influence to avoid byproduct formation, catalyst poisoning, and also to promote the reduction.^13,54^ At the same time, the presence of an excess of water might lead to the unwanted backward reaction and hydrolysis of the enamine intermediate.

**Fig. 2:**
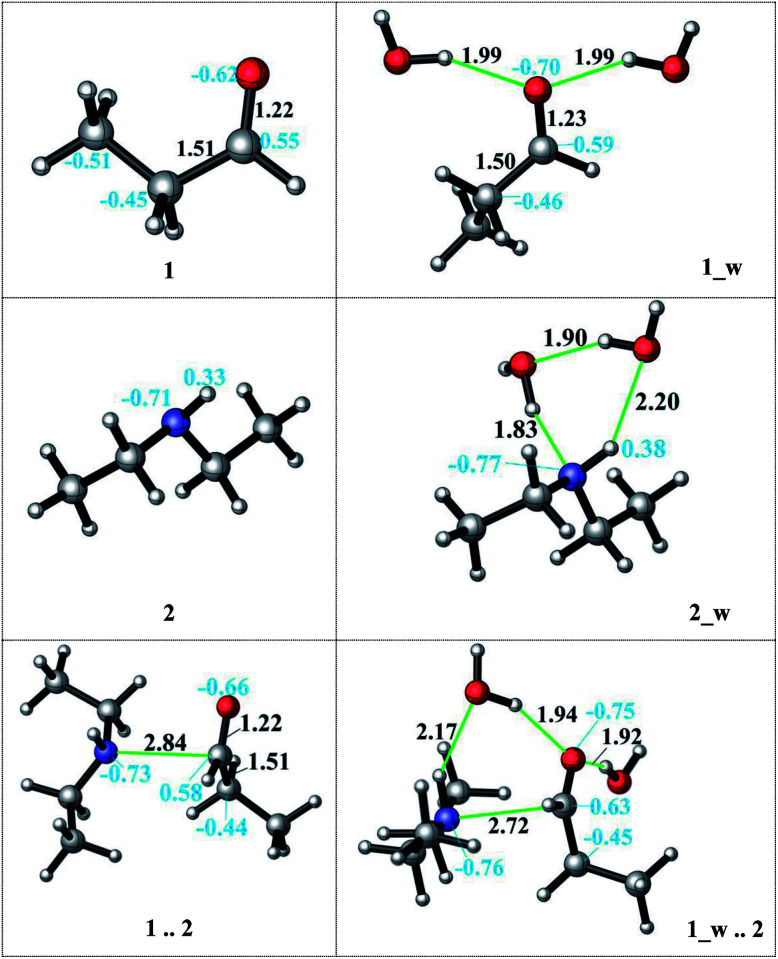
The effect of the water assistance on the pre-complex formation. (distances are given in Å, black; NBO charges are presented in blue).

The direct reductive amination reaction can be catalysed by non-aqueous hydrogen bonding solvents *e.g. via* thiourea coordination by means of the imine activation in the absence of an acid.^55^ Moreover, kinetic studies show that the added amount of the H-bond donor has no effect on the rate-determining step and is only needed in catalytic amounts. Additionally, the role of a hydrogen bond network has been shown to facilitate the nucleophilic addition and stabilize intermediates.^33,56^ A diethylamine and an acid complex was crystallized as an intermediate structure during a reductive amination which highlights the importance of the H-bond interactions during the reaction.^51^ We here investigate the catalytic effect and mechanistic role of explicitly coordinating water molecules on the kinetics (transition state barriers) and thermodynamics in the reductive amination. Several possible coordination modes of the water molecules *via* H-bonding were analysed to model the reaction medium. Initially, water coordination to the amine and to the aldehyde was established individually. First, we hypothesized that the H-bond stabilization of the amine is more likely due to its basic nature. The assistance of two explicit water molecules (complex 2_w, see Fig. 2) provides 9–4 kcal mol^−1^ extra stabilization to the amine in DMF and decane, respectively. The charge on the nitrogen atom changes (from −0.71 to −0.77) and water coordination thus increases the nucleophilicity of the nitrogen atom.

Then, solvation of propanal by two assisting water molecules which form hydrogen bonds with the oxygen lone pairs was investigated. This H-bonding stabilizes the initial aldehyde by 12–4 kcal mol^−1^ in DMF and decane, respectively. The charge of the reactive carbon atom changes from 0.55 to 0.59 by explicit water coordination, and thus the electrophilicity was promoted. After observing the favorable effect of the explicit H-bonding, we considered a full first solvation shell to connect the amine and the aldehyde by a detailed hydrogen bonding network of the water molecules. In our situation, however, such a water-mediated hydrogen bonding was not found to be feasible due to the hydrophobic character of the long carbon chains. Moreover, if a hydrogen-bonded cage-like structure was to be identified in quantum chemical calculations, it would be too minor and not be stable and persistent at experimental reaction conditions above a temperature of 100 °C.

**Scheme 4:**
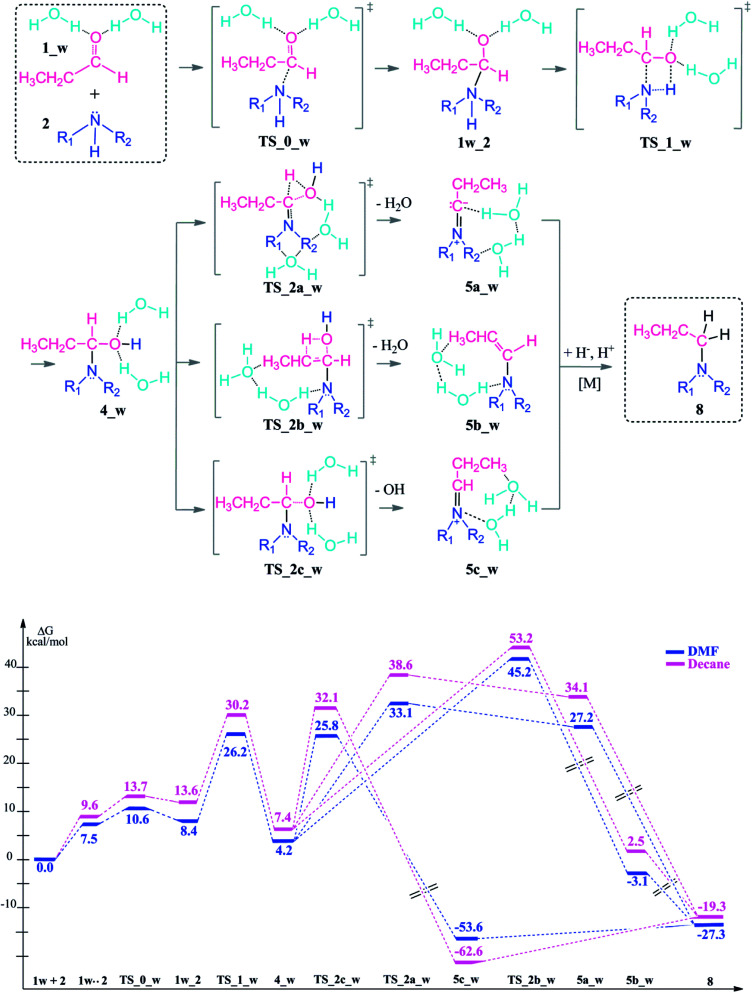
Reaction mechanism including transition states and intermediates and the free energy profile for reductive amination reaction with explicit water assistance, (R_1_ = R_2_ = –CH_2_CH_3_).

Although the water coordination increases the nucleophilicity of the amine, this coordination hinders the nucleophilic attack and a desolvation has to occur first. In case of aldehyde solvation, the water coordination has a positive effect on the charge density. One of the coordinating water molecules can occupy a bridging position between the amine (H-bond donor) and the aldehyde (H-bond acceptor) and facilitate the attack (Fig. 2, 1_w .. 2). Thus, the resulting aldehyde complex (1_w) was used as the starting structure (see Scheme 4) during the explicit solvent-assisted reaction. Subsequently, the entire reaction pathway including transition state localizations was elucidated in detail.

Upon hydrogen bonding, the electrophilicity of the carbonyl carbon atom in 1_w structure increases due to the increase in charge on the oxygen atom (from −0.62 to −0.70, see Fig. 2, top). In the aldehyde–amine pre-complex, this leads to an extra stabilization by 10.6–6 kcal mol^−1^ in DMF and decane due to the water molecule mediated interaction between aldehyde and amine (see Fig. 2, bottom). The pre-complex 1_w .. 2 is more tightly bound and the N⋯C distance decreases from 2.84 Å to 2.72 Å. Coordination by water molecules leads to a different stepwise nucleophile attack pathway which now proceeds *via*TS_0_w (amine addition) to give 1w_2 and *via*TS_1_w (proton transfer) to 4_w (see Scheme 4, top). Here, the nucleophilic addition proceeds in a stepwise fashion whereas in the absence of water it proceeded in a concerted fashion (TS_1, Scheme 3). Explicit water coordination lowers the barrier of activation by 5.5–9.9 kcal mol^−1^ which is in agreement with similar results obtained for one and two methanol molecules using DFT calculations.^33^ Also for the Schiff base formation, a decrease of energy barriers by inclusion of explicit waters was reported.^57,58^

After the formation of 4_w, the water elimination step by proton abstraction from the C_α_ carbon is facilitated by explicit water coordination. This solvent cage lowers the barrier of the condensation reaction, particularly for the transition state TS_2a_w (by 8–7.1 kcal mol^−1^) where one solvent water coordinates to the leaving water molecule. On the other hand, water coordination disfavours elimination from C_β_ of the enamine TS_2b_w by 0.1–5.8 kcal mol^−1^.

The dissociation of the –OH group by the assistance of the two water molecules (TS_2c_w) has an energy barrier of only 25.8–32.1 kcal mol^−1^ which is the lowest energy TS of all elimination possibilities. This reaction step is exergonic by 53.6–62.6 kcal mol^−1^ and results an iminium ion complex (5c_w) with two coordinating water molecules.

The introduction of the explicit solvation to the system effects also the energy barriers for the following water release during the condensation reaction. When solvent molecules are considered, water release *via* C_α_ proton abstraction is kinetically favored over C_β_ (TS_2a_w*vs.*TS_2b_w). However, the relative thermodynamic stabilities of the intermediates 5a, 5b, 5c*vs.*5a_w, 5b_w, 5c_w are preserved in neutral and water coordinated systems and in favor of the 5c and 5c_w formation.

We can thus show that the choice of an explicitly coordinating hydrogen-bonding solvent (here water) significantly changes the kinetic control of the reductive amination in that initial coupling step although it does not completely change the prefererred reaction pathway.

#### 3.2.3 The role of an acid as a co-catalyst

Experimentally, the presence of an acid as a co-catalyst was found to favorably influence the reductive amination reaction. For example, an optimum pH range of 2.5–3.5 in aqueous solution gives the best performance for cyclometallated iridium complexes in a formic acid/formate buffer solution.^14^ Different aspects of the role of the acid addition in the reductive amination reaction are discussed. Some suggest that the presence of a weak acid will facilitate and promote the first addition step whereas strongly acidic solutions would prevent further reactions of the initially formed primary amine by forming the ammonium salt.^13^ The pH value is particularly relevant for an optimization of the catalytic selectivity and the yield of the reaction. It was also shown that the pH level of the reaction media can affect the catalyst activity, its enantioselectivity and in particular the kinetics by a factor of six.^14^

We here investigate the role of an acid which acts as a co-catalyst in the reductive amination reaction at mildly acidic conditions (pH 4 or 5; for example acetic acid or formic acid). In order to rationalize the role of the acid as a co-catalysts, two scenarios were investigated.

**Scheme 5:**
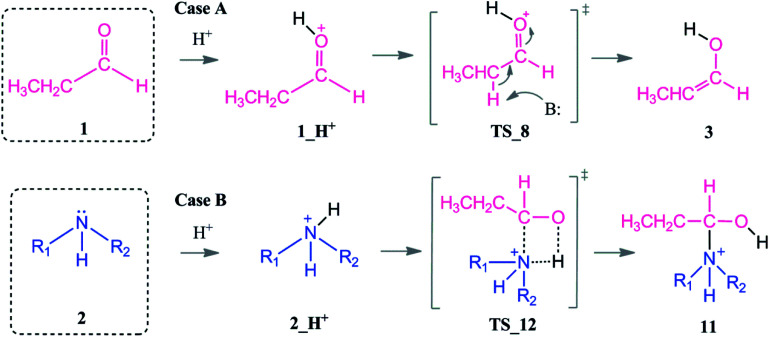
Coordination modes of an acid which acts as a co-catalyst *via* direct protonation of the aldehyde (Case A) and amine (Case B).

In the presence of an acid, protonation of both the aldehyde and amine are possible (Scheme 5). As a first case scenario, protonation of the aldehyde was investigated (Case A). Here, the carbonyl oxygen of the aldehyde is protonated (1_H^+^) and afterwards an alpha proton is abstracted (TS_8) by the amine (which was also considered as the base here) with a barrier of 9–3 kcal mol^−1^. As a result of this keto–enol tautomerization, the aldehyde is in equilibrium with its enol structure. The keto–enol equilibrium is known to be thermodynamically driven and usually favorable on the keto side. Thus, the aldehyde (1) is lower in energy by around 12 kcal mol^−1^ than the enol compound (3).

The protonation of the amine is examined as Case B. This step is feasible due to the basic character of the amine. In a recent study on reductive amination reaction with Rh complexes, it is suggested that this protonation of amine plays a key role in the catalytic cycle especially in the absence of an external hydrogen source.^59^ However, the nucleophilicity of the amine is abandoned by the formation of a quaternary ammonium compound (2_H^+^) which will disable the attack to the aldehyde. In this respect, we identified an intermolecular proton transfer from the ammonium compound to the carbonyl oxygen of the aldehyde. We successfully located the transition state (TS_12) for a concerted proton transfer and nucleophilic addition to the aldehyde. The proton affinity of the amine is around 43 kcal mol^−1^ higher than that of the aldehyde and thus the protonation of the amine (Case B) is thermodynamically more favorable.

However, besides the favorable thermodynamics upon protonation, the kinetic feasibility of the reaction after protonation of the reactants is still a question. First, we are investigating the reaction pathway following the enol formation. Then, the mechanism of the reaction *via* initially protonated structures (Case A *vs.* Case B) will be discussed in comparison.

#### 3.2.4 Reaction path following the keto–enol tautomerization

**Scheme 6:**
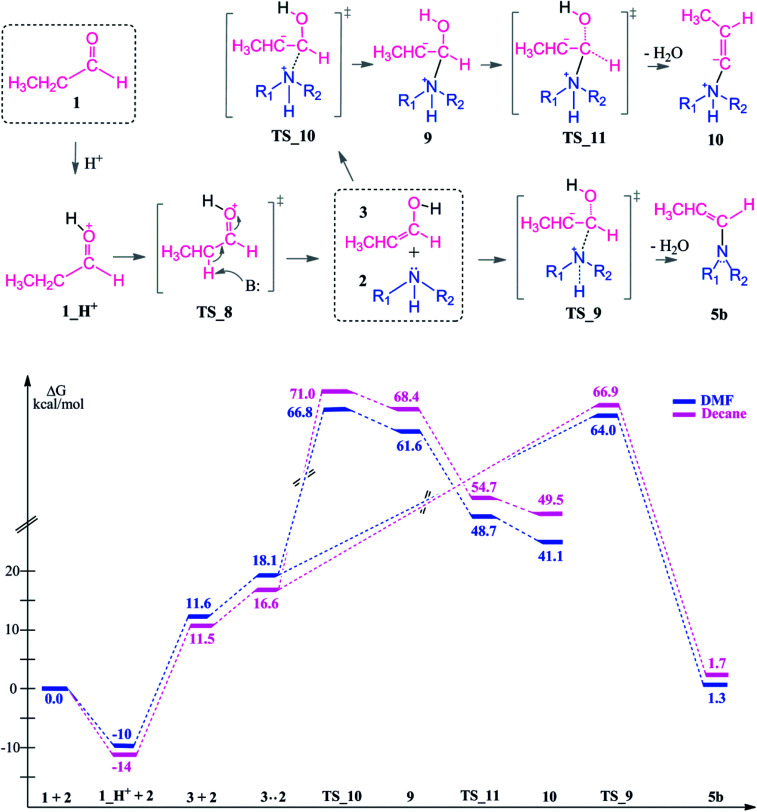
Reaction mechanism including identified transition states and intermediate structures and the free energy profile for reductive amination reaction following the keto–enol tautomerization, (R_1_ = R_2_ = –CH_2_CH_3_).

Protonation of the aldehyde (1_H^+^) is exothermic by 10–14 kcal mol^−1^. After the keto–enol tautomerization (see above), the reaction between the enol and the substrate diethylamine was investigated (Scheme 6). Two different pathways which both lead to the enamine intermediate can be distinguished. The first route is following the nucleophilic attack *via*TS_10 (>66 kcal mol^−1^) which then leads to the high energy adduct 9 which is 62–68 kcal mol^−1^ higher in energy than the substrates. From 9, C_α_ proton abstraction and water elimination may proceed. Here, the leaving –OH group is stabilized by intramolecular interactions with the nearby methyl hydrogen atoms of the alkyl chain. Then, elimination from the alpha position occurred *via*TS_11 to give the enamine zwitter ion 10 which is high in energy and inaccessible.

The second route is the nucleophilic addition of the amine and an intramolecular water elimination by abstraction of a proton from the positively charged nitrogen atom *via*TS_9. This transition state barrier is comparable to TS_10 (within 3–4 kcal mol^−1^) but the formation of the intermediate 5b is thermodynamically more favorable and it is significantly more stable compared to 10 by 40–48 kcal mol^−1^. After the formation of 5b, hydrogenation proceeds in analogy to the neutral pathway (Scheme 3) by first hydride binding to C_α_ or C_β_ and subsequent protonation of the remaining carbon atom.

**Fig. 3:**
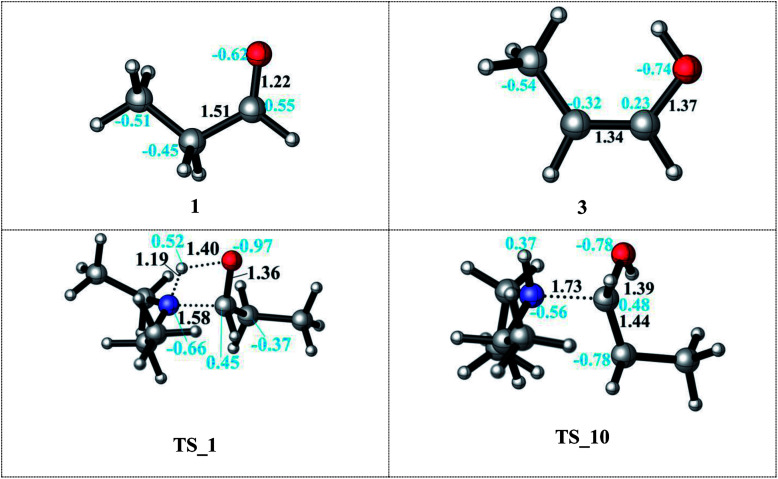
Details of the role of the acid co-catalyst in the nucleophilic attack of the amine. (Left) aldehyde substrate and TS_1 in neutral media. (Right) enol substrate and TS_10 in acidic media.

Protonation of the aldehyde and then formation of the enol significantly affects the charge distribution in the substrates (see Fig. 3). Protonation of the oxygen atom decreases the electrophilicity of the carbon atom from an atomic charge of 0.55 for the aldehyde to 0.23 for the enol. Thus, the reactivity of the carbon center is lowered and renders this reaction pathway inaccessible. This effect is also reflected in the activation barriers of this step (TS_10*vs.*TS_1) which show a difference of 32.4–36.9 kcal mol^−1^ and an elongation of the N⋯C distance from 1.58 to 1.73 Å in the transition state (see Fig. 3).

#### 3.2.5 Initial protonation of the substrates (Case A *vs.* Case B)

**Scheme 7:**
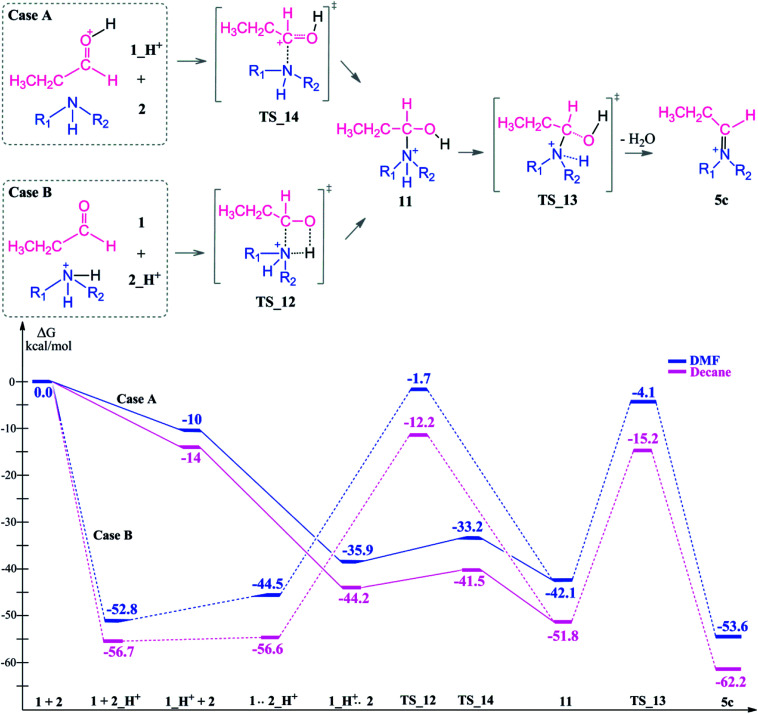
Reaction mechanism including transition states and reaction intermediate structures and the free energy profile for reductive amination reaction in the presence of an acid as a co-catalyst for Case A (full lines) and Case B (dashed lines), (R_1_ = R_2_ = –CH_2_CH_3_).

After initial protonation of the aldehyde (1_H^+^) mentioned as Case A, amine attack is also considered without enol formation taking place (Scheme 7). In this case, the protonated aldehyde and amine are approaching each other due to the enhanced electrophilicity of the carbonyl group. A pre-complex formation is exergonic by 36 to 42 kcal mol^−1^ and the following nucleophilic addition takes place *via*TS_14 with a small barrier of 3 kcal mol^−1^ only. The resulting intermediate 11 is around 10 kcal mol^−1^ lower than the transition state.

In TS_13, ^−^OH and a proton from the nitrogen are the leaving groups. Here, α and β eliminations are not feasible because of the acidic character of the ammonium proton. Thus, water elimination easily proceeds *via*TS_13 as the only distinct way. As a result of this elimination, the iminium intermediate 5c is obtained as the only candidate. Thus, the final reduction of intermediate 5c (*via* the same reduction procedure as applied in Scheme 3) is most likely to occur *via* this pathway and gives the final product 8.

Due to the basic character of the amine, initial protonation of the nitrogen in the presence of an acid occurs as mentioned (Case B). The calculated proton affinity to yield 2_H^+^ is around 53–57 kcal mol^−1^. After an intramolecular proton transfer to the carbonyl oxygen atom, the nucleophilicity of the amine is recovered. Then, the binding of the amine to the protonated aldehyde was investigated (see Scheme 7). This nucleophilic attack *via*TS_12 is thermodynamically possible although it has a higher transition state barrier than that of TS_14. The driving force here is the proton affinity of the amine (around 55 kcal mol^−1^). After the formation of intermediate 11, water elimination occurs in the same manner as discussed above.

Starting from the initially protonated reaction paths which are kinetically different, the nucleophilic additions are feasible. However, both paths are giving the same intermediate (11) in the end. Later, the reaction will go through 5c and yield a long chain amine as the final product.

Comparison of the neutral and acidic pathways indicates comparable transition state barrier heights (and thus similar microkinetics), although they do display strongly different thermodynamic profiles. In the presence of an acid as a co-catalyst, the reductive amination can proceed easily due to the enhanced thermodynamic stability of the iminium ion intermediate 5c. This extra stability is the thermodynamic driving force of the first reaction half before the final reduction step.

## 4. Conclusion

The mechanism of the reductive amination reaction between an aldehyde and a secondary amine to give a tertiary amine product was investigated in detail. This family of product is of great relevance to the pharmaceutical and chemical industries.^1,3^ The overall thermodynamics of the reductive amination reaction of an aldehyde was found to be exergonic by around −15 kcal mol^−1^, independent of the carbon chain length and consideration of an organic solvent environment has a negligible effect on the free energy of the reaction.

The influence and control of the reaction by solvent properties (neutral, aqueous media, and in the presence of an acid as a co-catalyst) were investigated in detail including many different branching points.

In neutral media, the dissociation of a hydroxide ion after nucleophilic addition of the amine is clearly the preferred path and leads to an iminium as an intermediate. This is then easily hydrogenated to give the final product. In the presence of excess water, which is also released during the condensation step, the first part of the reaction is strongly affected. The formation of hydrogen bonds by explicit water coordination is increasing the electrophilicity of the aldehyde. Thus, the activation barrier for the nucleophilic addition is significantly lower and the formerly concerted amine addition becomes stepwise leading to lower energy barriers. Explicit water assistance drives the reaction pathway from an enamine- to an iminium ion-based intermediate and controls the total kinetics of the reaction process. Such insight is of relevance for the design of a suitable solvent in a large-scale production of tertiary amines from long-chain substrates from sustainable sources and the continuous removal of water during the process in order to minimize product loss due to hydrolysis.

Addition of an acid as a co-catalyst affects the entire reaction cycle, both the addition and hydrogenation steps. Keto–enol tautomerization *via* direct protonation of the aldehyde (Case A) can be ruled out since the formed enol has different electronic properties and loses its electrophilicity. The presence of an acid here increases the activation barrier and decelerates the rate of the reaction.

However, when first the amine is protonated (Case B), the entire pathway is significantly affected by an extra stabilization resulting from the amine's proton affinity. This protonation does not alter the barrier heights and thus the kinetics of the reaction are unchanged. However, the thermodynamic profile becomes more exothermic compared to the neutral pathway and clearly renders this pathway to become the preferred one. Thus, the presence of an acid as a co-catalyst strongly enhances the relative thermodynamic stability of the intermediate 5c and drives the reaction forward.

In conclusion, explicit coordination of solvent water and the co-catalyst acid assistance provide extra stabilization to different intermediates of the reductive amination of aldehydes with diethylamine and allow to guide the reaction along different pathways. Thus, by choice of solvent and pH, the microkinetics of different steps will strongly be affected and allow the control of the kinetics and thermodynamics of the ‘rate determining state’. So far, experiment did not fully resolve the structural, kinetic and thermodynamic effects of the addition of acids, bases, or water on the activity and selectivity of the catalysts.^13^ This computational work, for the first time, provides a detailed insight into these process control parameters and will aid the design of an appropriate reaction medium as well as a suitable catalyst for the final hydrogenation step. The choice of an appropriate transition metal-based catalyst to replace an organic hydride donor is the focus of currently ongoing work.

## Conflicts of interest

There are no conflicts to declare.

## Supplementary Material

RA-008-C8RA08135B-s001
