# Supplementary material for: Computational investigation of the control of the thermodynamics and microkinetics of the reductive amination reaction by solvent coordination and a co-catalyst
Source: RSC Adv. 2018 Oct 30;8(64):36662–74. doi: 10.1039/c8ra08135b (PMC9088820; doi:10.1039/c8ra08135b)
Supplement: RA-008-C8RA08135B-s001 [file RA-008-C8RA08135B-s001.pdf]

Electronic Supplementary Material (ESI) for RSC Advances.
This journal is © The Royal Society of Chemistry 2018

Supporting Information

Computational Investigation of the Control of Thermodynamics and Microkinetics of the Reductive Amination Reaction by Solvent Coordination and a Co-Catalyst

Esra Boz,[1,2] Nurcan Ş. Tüzün,[2] and Matthias Stein[1*]

[1]Max Planck Institute for Dynamics of Complex Technical Systems, Molecular Simulations and Design Group, Magdeburg, Germany
[2]Istanbul Technical University, Department of Chemistry, Istanbul, Turkey

**Corresponding Author: Dr Matthias Stein. matthias.stein@mpi-magdeburg.mpg.de*

Contents

Selected Bond Lengths and NBO Charges for the Compounds **2**

Figure S1. The selected bond lengths (in Å, black) and NBO charges (blue) of the transition state and intermediate structures for reductive amination reaction in neutral media. **2**

Figure S2. The selected bond lengths (in Å, black) and NBO charges (blue) of the transition state and intermediate structures for reductive amination reaction in neutral media with explicit water assistance. **3**

Figure S3. The selected bond lengths (in Å, black) and NBO charges (blue) of the transition state and intermediate structures for reductive amination reaction in low pH. **4**

Table S1. Relative Gibbs free energies of the transition states and intermediate structures for reductive amination reaction in neutral media calculated in the gas phase and implicit solvent (in DMF and decane). **5**

Table S2. Relative Gibbs free energies of the transition states and intermediate structures for reductive amination reaction in neutral media with explicit water assistance calculated in the gas phase and implicit solvent (in DMF and decane). **5**

Table S3. Relative Gibbs free energies of the transition states and intermediate structures for reductive amination reaction in the presence of an acid as a co-catalyst for Case A calculated in the gas phase and implicit solvent (in DMF and decane). **6**

Table S4. Relative Gibbs free energies of the transition states and intermediate structures for reductive amination reaction in the presence of an acid as a co-catalyst for Case B calculated in the gas phase and implicit solvent (in DMF and decane). **6**

Selected Bond Lengths and NBO Charges for the Compounds

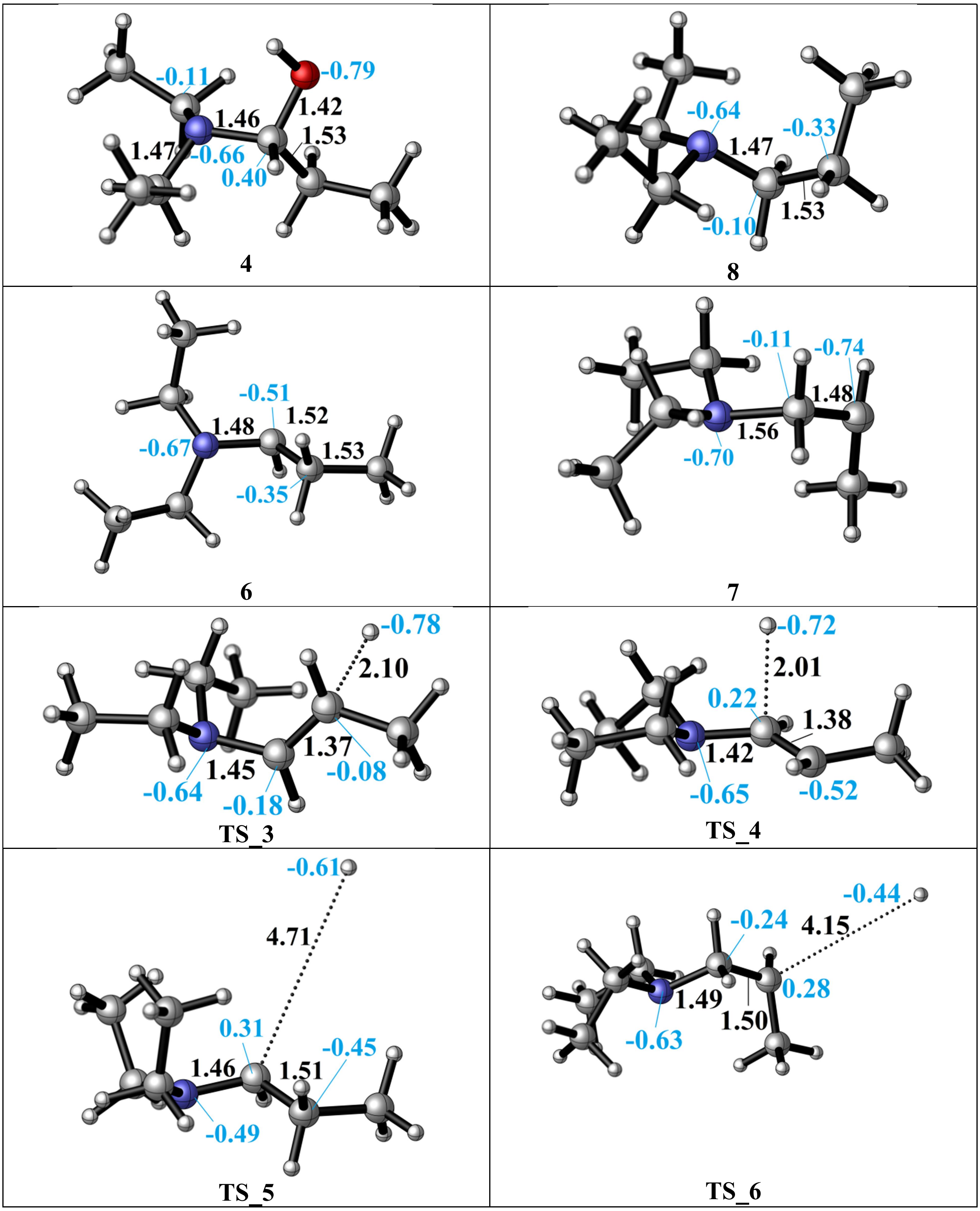

Figure S1. The selected bond lengths (in Å, black) and NBO charges (blue) of the transition state and intermediate structures for reductive amination reaction in neutral media.

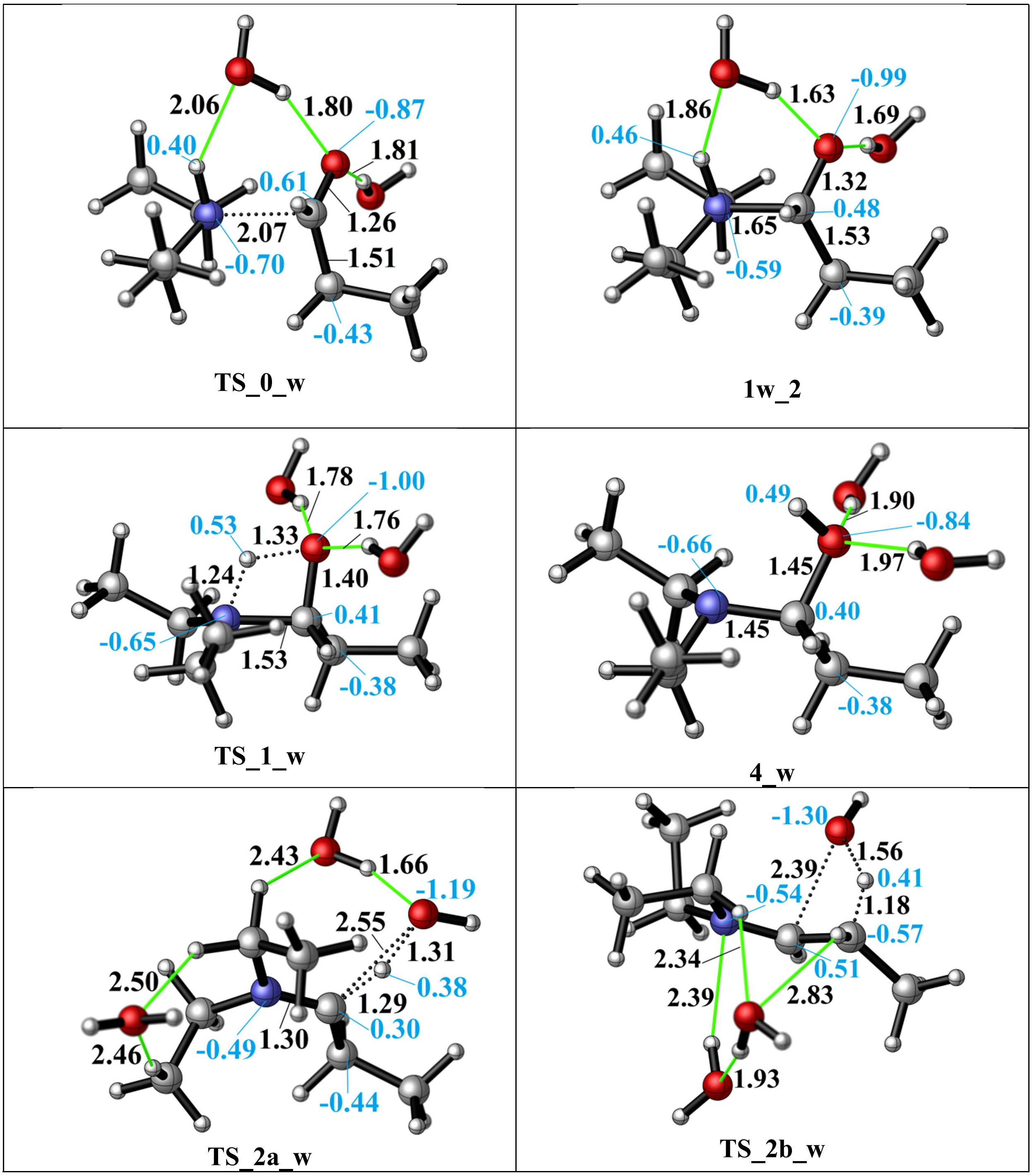

Figure S2. The selected bond lengths (in Å, black) and NBO charges (blue) of the transition state and intermediate structures for reductive amination reaction in neutral media with explicit water assistance.

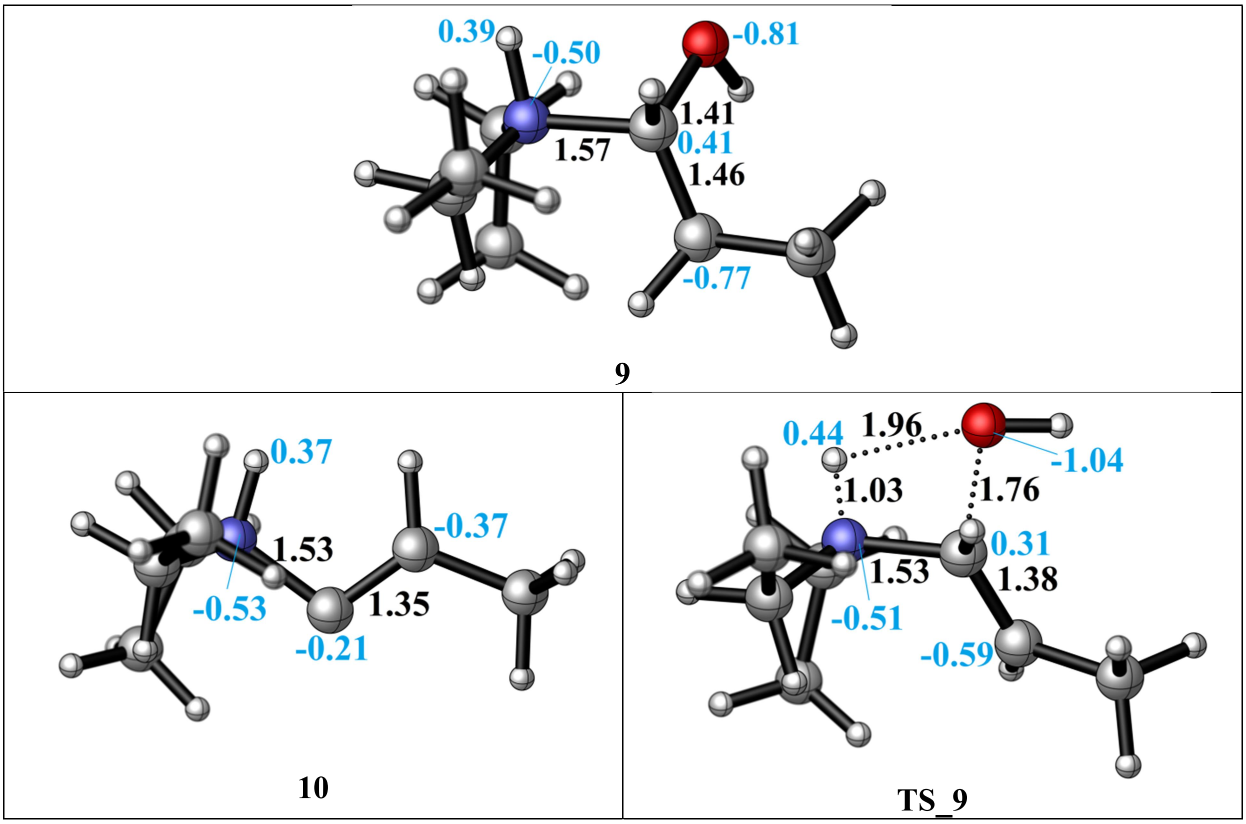

Figure S3. The selected bond lengths (in Å, black) and NBO charges (blue) of the transition state and intermediate structures for reductive amination reaction in low pH.

	1 .. 2	TS_1	4	TS_2a	TS_2b	5a	5b	5c	TS_3	TS_4	6	7	TS_5	TS_6	TS_7	8
**Gas phase**	4.2	33.3	2.1	48.9	48.9	33.4	2.1	-71.3	0.2	-1.2	-28.1	-25.9	-134.9	-125.2	-46.9	-14.7
**DMF**	9.1	32.4	5.6	41.1	45.1	26.8	1.3	-53.6	29.4	28.2	11.4	12.1	-6.7	38.0	-150	-15.1
**Decane**	7.6	34.1	3.7	45.7	47.4	30.7	1.7	-62.2	19.2	17.8	-5.8	-4.7	-45.7	-11.5	-74.9	-15.3

Table S1. Relative Gibbs free energies of the transition states and intermediate structures for reductive amination reaction in neutral media calculated in the gas phase and implicit solvent (in DMF and decane).

	1_w..2	TS_0_w	1w_2	TS_1_w	4_w	TS_2a_w	TS_2b_w	TS_2c_w	5a_w	5b_w	5c_w	8
**Gas phase**	2.5	7.3	8.6	24.3	0.9	34.0	49.6	28.1	30.4	-2.1	-81.8	-21.5
**DMF**	7.5	10.6	8.4	26.2	4.2	33.1	45.2	25.8	27.2	-3.1	-53.6	-27.3
**Decane**	9.6	13.7	13.6	30.2	7.4	38.6	53.2	32.1	34.1	2.5	-62.6	-19.3

Table S2. Relative Gibbs free energies of the transition states and intermediate structures for reductive amination reaction in neutral media with explicit water assistance calculated in the gas phase and implicit solvent (in DMF and decane).

	1_H^+ .. 2	3+2	3 .. 2	TS_10	9	TS_11	10	TS_9	5b
**Gas phase**	-19.0	11.1	15.5	72.5	71.4	58.0	54.0	68.5	2.2
**DMF**	-10	11.6	18.1	66.8	61.6	48.7	41.1	64.0	1.7
**Decane**	-14	11.5	16.6	71.0	68.4	54.7	49.5	66.9	1.3

Table S3. Relative Gibbs free energies of the transition states and intermediate structures for reductive amination reaction following the keto-enol tautomerization in the presence of an acid calculated in the gas phase and implicit solvent (in DMF and decane).

	1 + 2_H^+	1 .. 2_H^+	TS_12	11	TS_13	TS_14	5c
**Gas phase**	-61.6	-69.2	-24.0	-62.9	-27.1	-51.9	-71.3
**DMF**	-52.8	-44.5	-1.7	-42.1	-4.1	-33.2	-53.6
**Decane**	-56.7	-56.6	-12.2	-51.8	-15.2	-41.5	-62.2

Table S4. Relative Gibbs free energies of the transition states and intermediate structures for reductive amination reaction in the presence of an acid as a co-catalyst for Case A and Case B calculated in the gas phase and implicit solvent (in DMF and decane).

Free energies and thermal corrections of the transition states and intermediate structures for reductive amination reaction

1

```
Zero-point correction=                           0.085349
(Hartree/Particle)
 Thermal correction to Energy=                    0.090302
 Thermal correction to Enthalpy=                  0.091247
 Thermal correction to Gibbs Free Energy=         0.058093
 Sum of electronic and zero-point Energies=           -192.560172
 Sum of electronic and thermal Energies=              -192.555218
 Sum of electronic and thermal Enthalpies=            -192.554274
 Sum of electronic and thermal Free Energies=         -192.587428
```

2

```
Zero-point correction=                           0.151522
(Hartree/Particle)
 Thermal correction to Energy=                    0.158312
 Thermal correction to Enthalpy=                  0.159257
 Thermal correction to Gibbs Free Energy=         0.121372
 Sum of electronic and zero-point Energies=           -213.022772
 Sum of electronic and thermal Energies=              -213.015982
 Sum of electronic and thermal Enthalpies=            -213.015038
 Sum of electronic and thermal Free Energies=         -213.052922
```

1..2

```
Zero-point correction=                           0.237742
(Hartree/Particle)
 Thermal correction to Energy=                    0.251508
 Thermal correction to Enthalpy=                  0.252452
 Thermal correction to Gibbs Free Energy=         0.192565
 Sum of electronic and zero-point Energies=           -405.588531
 Sum of electronic and thermal Energies=              -405.574764
 Sum of electronic and thermal Enthalpies=            -405.573820
 Sum of electronic and thermal Free Energies=         -405.633707
```

4

```
 Zero-point correction=                           0.241899
(Hartree/Particle)
 Thermal correction to Energy=                    0.253594
 Thermal correction to Enthalpy=                  0.254538
 Thermal correction to Gibbs Free Energy=         0.204816
 Sum of electronic and zero-point Energies=           -405.599881
 Sum of electronic and thermal Energies=              -405.588187
 Sum of electronic and thermal Enthalpies=            -405.587242
```

Sum of electronic and thermal Free Energies= -405.636965

5a

Zero-point correction= 0.212529
(Hartree/Particle)
Thermal correction to Energy= 0.222896
Thermal correction to Enthalpy= 0.223840
Thermal correction to Gibbs Free Energy= 0.176943
Sum of electronic and zero-point Energies= -329.280828
Sum of electronic and thermal Energies= -329.270461
Sum of electronic and thermal Enthalpies= -329.269517
Sum of electronic and thermal Free Energies= -329.316414

5b

Zero-point correction= 0.212816
(Hartree/Particle)
Thermal correction to Energy= 0.223103
Thermal correction to Enthalpy= 0.224047
Thermal correction to Gibbs Free Energy= 0.177874
Sum of electronic and zero-point Energies= -329.331386
Sum of electronic and thermal Energies= -329.321099
Sum of electronic and thermal Enthalpies= -329.320155
Sum of electronic and thermal Free Energies= -329.366328

5c

Zero-point correction= 0.227197
(Hartree/Particle)
Thermal correction to Energy= 0.237652
Thermal correction to Enthalpy= 0.238597
Thermal correction to Gibbs Free Energy= 0.191530
Sum of electronic and zero-point Energies= -329.709831
Sum of electronic and thermal Energies= -329.699375
Sum of electronic and thermal Enthalpies= -329.698431
Sum of electronic and thermal Free Energies= -329.745497

6

Zero-point correction= 0.218929
(Hartree/Particle)
Thermal correction to Energy= 0.229676
Thermal correction to Enthalpy= 0.230620
Thermal correction to Gibbs Free Energy= 0.182968
Sum of electronic and zero-point Energies= -329.871368
Sum of electronic and thermal Energies= -329.860621
Sum of electronic and thermal Enthalpies= -329.859677
Sum of electronic and thermal Free Energies= -329.907330

7

```
Zero-point correction=                           0.218630
(Hartree/Particle)
 Thermal correction to Energy=                    0.229321
 Thermal correction to Enthalpy=                  0.230265
 Thermal correction to Gibbs Free Energy=         0.182886
 Sum of electronic and zero-point Energies=           -329.868127
 Sum of electronic and thermal Energies=              -329.857436
 Sum of electronic and thermal Enthalpies=            -329.856492
 Sum of electronic and thermal Free Energies=         -329.903871
```

8

```
Zero-point correction=                           0.236890
(Hartree/Particle)
 Thermal correction to Energy=                    0.247539
 Thermal correction to Enthalpy=                  0.248484
 Thermal correction to Gibbs Free Energy=         0.200831
 Sum of electronic and zero-point Energies=           -330.518445
 Sum of electronic and thermal Energies=              -330.507795
 Sum of electronic and thermal Enthalpies=            -330.506850
 Sum of electronic and thermal Free Energies=         -330.554504
```

TS_1

```
Zero-point correction=                           0.237190
(Hartree/Particle)
 Thermal correction to Energy=                    0.248659
 Thermal correction to Enthalpy=                  0.249603
 Thermal correction to Gibbs Free Energy=         0.199818
 Sum of electronic and zero-point Energies=           -405.546564
 Sum of electronic and thermal Energies=              -405.535095
 Sum of electronic and thermal Enthalpies=            -405.534151
 Sum of electronic and thermal Free Energies=         -405.583936
```

TS_2a

```
Zero-point correction=                           0.235933
(Hartree/Particle)
 Thermal correction to Energy=                    0.248488
 Thermal correction to Enthalpy=                  0.249432
 Thermal correction to Gibbs Free Energy=         0.197913
 Sum of electronic and zero-point Energies=           -405.524475
 Sum of electronic and thermal Energies=              -405.511921
 Sum of electronic and thermal Enthalpies=            -405.510976
 Sum of electronic and thermal Free Energies=         -405.562496
```

TS_2b

```
Zero-point correction=                           0.235547
(Hartree/Particle)
 Thermal correction to Energy=                    0.247675
 Thermal correction to Enthalpy=                  0.248620
 Thermal correction to Gibbs Free Energy=         0.198201
 Sum of electronic and zero-point Energies=           -405.525038
 Sum of electronic and thermal Energies=              -405.512910
 Sum of electronic and thermal Enthalpies=            -405.511965
 Sum of electronic and thermal Free Energies=         -405.562384
```

TS_3

```
Zero-point correction=                           0.212840
(Hartree/Particle)
 Thermal correction to Energy=                    0.223670
 Thermal correction to Enthalpy=                  0.224615
 Thermal correction to Gibbs Free Energy=         0.177393
 Sum of electronic and zero-point Energies=           -329.826768
 Sum of electronic and thermal Energies=              -329.815938
 Sum of electronic and thermal Enthalpies=            -329.814994
 Sum of electronic and thermal Free Energies=         -329.862215
```

TS_4

```
Zero-point correction=                           0.213594
(Hartree/Particle)
 Thermal correction to Energy=                    0.224274
 Thermal correction to Enthalpy=                  0.225218
 Thermal correction to Gibbs Free Energy=         0.178328
 Sum of electronic and zero-point Energies=           -329.829301
 Sum of electronic and thermal Energies=              -329.818620
 Sum of electronic and thermal Enthalpies=            -329.817676
 Sum of electronic and thermal Free Energies=         -329.864567
```

TS_5

```
Zero-point correction=                           0.222958
(Hartree/Particle)
 Thermal correction to Energy=                    0.235123
 Thermal correction to Enthalpy=                  0.236067
 Thermal correction to Gibbs Free Energy=         0.185221
 Sum of electronic and zero-point Energies=           -330.302066
 Sum of electronic and thermal Energies=              -330.289901
 Sum of electronic and thermal Enthalpies=            -330.288957
 Sum of electronic and thermal Free Energies=         -330.339803
```

TS_6

```
Zero-point correction=                           0.222299
(Hartree/Particle)
 Thermal correction to Energy=                    0.234377
 Thermal correction to Enthalpy=                  0.235322
 Thermal correction to Gibbs Free Energy=         0.184221
 Sum of electronic and zero-point Energies=           -330.286258
 Sum of electronic and thermal Energies=              -330.274179
 Sum of electronic and thermal Enthalpies=            -330.273235
 Sum of electronic and thermal Free Energies=         -330.324335
```

TS_7

```
Zero-point correction=                           0.222964
(Hartree/Particle)
 Thermal correction to Energy=                    0.235039
 Thermal correction to Enthalpy=                  0.235984
 Thermal correction to Gibbs Free Energy=         0.184901
 Sum of electronic and zero-point Energies=           -330.305654
 Sum of electronic and thermal Energies=              -330.293578
 Sum of electronic and thermal Enthalpies=            -330.292634
 Sum of electronic and thermal Free Energies=         -330.343717
```

1_w

```
Zero-point correction=                           0.134452
(Hartree/Particle)
 Thermal correction to Energy=                    0.146576
 Thermal correction to Enthalpy=                  0.147520
 Thermal correction to Gibbs Free Energy=         0.094394
 Sum of electronic and zero-point Energies=           -345.077930
 Sum of electronic and thermal Energies=              -345.065807
 Sum of electronic and thermal Enthalpies=            -345.064863
 Sum of electronic and thermal Free Energies=         -345.117989
```

1w..2

```
Zero-point correction=                           0.288043
(Hartree/Particle)
 Thermal correction to Energy=                    0.308158
 Thermal correction to Enthalpy=                  0.309103
 Thermal correction to Gibbs Free Energy=         0.236771
 Sum of electronic and zero-point Energies=           -558.115591
 Sum of electronic and thermal Energies=              -558.095475
 Sum of electronic and thermal Enthalpies=            -558.094531
 Sum of electronic and thermal Free Energies=         -558.166862
```

1w_2

```
Zero-point correction=                           0.292837
(Hartree/Particle)
 Thermal correction to Energy=                    0.310149
 Thermal correction to Enthalpy=                  0.311093
 Thermal correction to Gibbs Free Energy=         0.247712
 Sum of electronic and zero-point Energies=           -558.112066
 Sum of electronic and thermal Energies=              -558.094755
 Sum of electronic and thermal Enthalpies=            -558.093811
 Sum of electronic and thermal Free Energies=         -558.157192
```

4_w

```
Zero-point correction=                           0.291361
(Hartree/Particle)
 Thermal correction to Energy=                    0.309871
 Thermal correction to Enthalpy=                  0.310815
 Thermal correction to Gibbs Free Energy=         0.244154
 Sum of electronic and zero-point Energies=           -558.122304
 Sum of electronic and thermal Energies=              -558.103795
 Sum of electronic and thermal Enthalpies=            -558.102850
 Sum of electronic and thermal Free Energies=         -558.169512
```

5a_w

```
Zero-point correction=                           0.260838
(Hartree/Particle)
 Thermal correction to Energy=                    0.278627
 Thermal correction to Enthalpy=                  0.279571
 Thermal correction to Gibbs Free Energy=         0.213730
 Sum of electronic and zero-point Energies=           -481.804653
 Sum of electronic and thermal Energies=              -481.786863
 Sum of electronic and thermal Enthalpies=            -481.785919
 Sum of electronic and thermal Free Energies=         -481.851760
```

5b_w

```
Zero-point correction=                           0.263056
(Hartree/Particle)
 Thermal correction to Energy=                    0.279499
 Thermal correction to Enthalpy=                  0.280444
 Thermal correction to Gibbs Free Energy=         0.218939
 Sum of electronic and zero-point Energies=           -481.859412
 Sum of electronic and thermal Energies=              -481.842968
 Sum of electronic and thermal Enthalpies=            -481.842024
 Sum of electronic and thermal Free Energies=         -481.903528
```

5c_w

```
Zero-point correction=                          0.276565
(Hartree/Particle)
 Thermal correction to Energy=                   0.293813
 Thermal correction to Enthalpy=                 0.294757
 Thermal correction to Gibbs Free Energy=        0.229173
 Sum of electronic and zero-point Energies=          -482.245461
 Sum of electronic and thermal Energies=             -482.228214
 Sum of electronic and thermal Enthalpies=           -482.227269
 Sum of electronic and thermal Free Energies=        -482.292853
```

TS_0_w

```
Zero-point correction=                          0.290158
(Hartree/Particle)
 Thermal correction to Energy=                   0.308202
 Thermal correction to Enthalpy=                 0.309146
 Thermal correction to Gibbs Free Energy=        0.243226
 Sum of electronic and zero-point Energies=          -558.112346
 Sum of electronic and thermal Energies=             -558.094302
 Sum of electronic and thermal Enthalpies=           -558.093357
 Sum of electronic and thermal Free Energies=        -558.159278
```

TS_1_w

```
Zero-point correction=                          0.287629
(Hartree/Particle)
 Thermal correction to Energy=                   0.305228
 Thermal correction to Enthalpy=                 0.306172
 Thermal correction to Gibbs Free Energy=        0.241545
 Sum of electronic and zero-point Energies=          -558.086110
 Sum of electronic and thermal Energies=             -558.068511
 Sum of electronic and thermal Enthalpies=           -558.067567
 Sum of electronic and thermal Free Energies=        -558.132194
```

TS_2a_w

```
Zero-point correction=                          0.282535
(Hartree/Particle)
 Thermal correction to Energy=                   0.302110
 Thermal correction to Enthalpy=                 0.303054
 Thermal correction to Gibbs Free Energy=        0.232475
 Sum of electronic and zero-point Energies=          -558.066631
 Sum of electronic and thermal Energies=             -558.047056
 Sum of electronic and thermal Enthalpies=           -558.046112
 Sum of electronic and thermal Free Energies=        -558.116691
```

TS_2b_w

```
Zero-point correction=                           0.284207
(Hartree/Particle)
 Thermal correction to Energy=                    0.303455
 Thermal correction to Enthalpy=                  0.304399
 Thermal correction to Gibbs Free Energy=         0.236576
 Sum of electronic and zero-point Energies=          -558.044193
 Sum of electronic and thermal Energies=             -558.024945
 Sum of electronic and thermal Enthalpies=           -558.024001
 Sum of electronic and thermal Free Energies=        -558.091824
```

TS_2c_w

```
Zero-point correction=                           0.289165
(Hartree/Particle)
 Thermal correction to Energy=                    0.306285
 Thermal correction to Enthalpy=                  0.307229
 Thermal correction to Gibbs Free Energy=         0.245228
 Sum of electronic and zero-point Energies=          -558.082204
 Sum of electronic and thermal Energies=             -558.065084
 Sum of electronic and thermal Enthalpies=           -558.064140
 Sum of electronic and thermal Free Energies=        -558.126141
```

1_H+

```
Zero-point correction=                           0.098501
(Hartree/Particle)
 Thermal correction to Energy=                    0.103597
 Thermal correction to Enthalpy=                  0.104541
 Thermal correction to Gibbs Free Energy=         0.071127
 Sum of electronic and zero-point Energies=          -192.852571
 Sum of electronic and thermal Energies=             -192.847475
 Sum of electronic and thermal Enthalpies=           -192.846530
 Sum of electronic and thermal Free Energies=        -192.879945
```

3

```
Zero-point correction=                           0.085485
(Hartree/Particle)
 Thermal correction to Energy=                    0.090659
 Thermal correction to Enthalpy=                  0.091603
 Thermal correction to Gibbs Free Energy=         0.057792
 Sum of electronic and zero-point Energies=          -192.541993
 Sum of electronic and thermal Energies=             -192.536819
 Sum of electronic and thermal Enthalpies=           -192.535875
 Sum of electronic and thermal Free Energies=        -192.569686
```

3..2

```
Zero-point correction=                           0.238010
(Hartree/Particle)
 Thermal correction to Energy=                    0.252007
 Thermal correction to Enthalpy=                  0.252951
 Thermal correction to Gibbs Free Energy=         0.193581
 Sum of electronic and zero-point Energies=           -405.571272
 Sum of electronic and thermal Energies=              -405.557276
 Sum of electronic and thermal Enthalpies=            -405.556332
 Sum of electronic and thermal Free Energies=         -405.615701
```

9

```
Zero-point correction=                           0.241288
(Hartree/Particle)
 Thermal correction to Energy=                    0.252716
 Thermal correction to Enthalpy=                  0.253660
 Thermal correction to Gibbs Free Energy=         0.204910
 Sum of electronic and zero-point Energies=           -405.490156
 Sum of electronic and thermal Energies=              -405.478729
 Sum of electronic and thermal Enthalpies=            -405.477785
 Sum of electronic and thermal Free Energies=         -405.526534
```

10

```
Zero-point correction=                           0.212675
(Hartree/Particle)
 Thermal correction to Energy=                    0.223006
 Thermal correction to Enthalpy=                  0.223950
 Thermal correction to Gibbs Free Energy=         0.177513
 Sum of electronic and zero-point Energies=           -329.248498
 Sum of electronic and thermal Energies=              -329.238166
 Sum of electronic and thermal Enthalpies=            -329.237222
 Sum of electronic and thermal Free Energies=         -329.283659
```

TS_8

```
Zero-point correction=                           0.250819
(Hartree/Particle)
 Thermal correction to Energy=                    0.263702
 Thermal correction to Enthalpy=                  0.264646
 Thermal correction to Gibbs Free Energy=         0.209943
 Sum of electronic and zero-point Energies=           -405.897342
 Sum of electronic and thermal Energies=              -405.884460
 Sum of electronic and thermal Enthalpies=            -405.883516
 Sum of electronic and thermal Free Energies=         -405.938219
```

TS_9

```
 Zero-point correction=                           0.239328
(Hartree/Particle)
 Thermal correction to Energy=                    0.251114
 Thermal correction to Enthalpy=                  0.252058
 Thermal correction to Gibbs Free Energy=         0.202321
 Sum of electronic and zero-point Energies=           -405.494122
 Sum of electronic and thermal Energies=              -405.482336
 Sum of electronic and thermal Enthalpies=            -405.481392
 Sum of electronic and thermal Free Energies=         -405.531128
```

TS_10

```
Zero-point correction=                           0.239770
(Hartree/Particle)
 Thermal correction to Energy=                    0.251137
 Thermal correction to Enthalpy=                  0.252081
 Thermal correction to Gibbs Free Energy=         0.202996
 Sum of electronic and zero-point Energies=           -405.488090
 Sum of electronic and thermal Energies=              -405.476723
 Sum of electronic and thermal Enthalpies=            -405.475779
 Sum of electronic and thermal Free Energies=         -405.524863
```

TS_11

```
 Zero-point correction=                           0.234270
(Hartree/Particle)
 Thermal correction to Energy=                    0.246693
 Thermal correction to Enthalpy=                  0.247637
 Thermal correction to Gibbs Free Energy=         0.196589
 Sum of electronic and zero-point Energies=           -405.510237
 Sum of electronic and thermal Energies=              -405.497815
 Sum of electronic and thermal Enthalpies=            -405.496871
 Sum of electronic and thermal Free Energies=         -405.547918
```

2_H+

```
Zero-point correction=                           0.166872
(Hartree/Particle)
 Thermal correction to Energy=                    0.173773
 Thermal correction to Enthalpy=                  0.174717
 Thermal correction to Gibbs Free Energy=         0.136558
 Sum of electronic and zero-point Energies=           -213.383006
 Sum of electronic and thermal Energies=              -213.376106
 Sum of electronic and thermal Enthalpies=            -213.375161
 Sum of electronic and thermal Free Energies=         -213.413320
```

1.. 2_H+

```
Zero-point correction=                           0.253744
(Hartree/Particle)
 Thermal correction to Energy=                    0.267427
 Thermal correction to Enthalpy=                  0.268372
 Thermal correction to Gibbs Free Energy=         0.209590
 Sum of electronic and zero-point Energies=           -405.968589
 Sum of electronic and thermal Energies=              -405.954905
 Sum of electronic and thermal Enthalpies=            -405.953961
 Sum of electronic and thermal Free Energies=         -406.012743
```

1_H+..2

```
Zero-point correction=                           0.257920
(Hartree/Particle)
 Thermal correction to Energy=                    0.269274
 Thermal correction to Enthalpy=                  0.270219
 Thermal correction to Gibbs Free Energy=         0.221868
 Sum of electronic and zero-point Energies=           -405.953070
 Sum of electronic and thermal Energies=              -405.941715
 Sum of electronic and thermal Enthalpies=            -405.940771
 Sum of electronic and thermal Free Energies=         -405.989122
```

11

```
Zero-point correction=                           0.256815
(Hartree/Particle)
 Thermal correction to Energy=                    0.268761
 Thermal correction to Enthalpy=                  0.269705
 Thermal correction to Gibbs Free Energy=         0.219508
 Sum of electronic and zero-point Energies=           -405.965383
 Sum of electronic and thermal Energies=              -405.953438
 Sum of electronic and thermal Enthalpies=            -405.952494
 Sum of electronic and thermal Free Energies=         -406.002691
```

TS_12

```
Zero-point correction=                           0.252168
(Hartree/Particle)
 Thermal correction to Energy=                    0.264896
 Thermal correction to Enthalpy=                  0.265841
 Thermal correction to Gibbs Free Energy=         0.210936
 Sum of electronic and zero-point Energies=           -405.899577
 Sum of electronic and thermal Energies=              -405.886848
 Sum of electronic and thermal Enthalpies=            -405.885904
 Sum of electronic and thermal Free Energies=         -405.940809
```

TS_13

```
Zero-point correction=                           0.250629
(Hartree/Particle)
 Thermal correction to Energy=                    0.262062
 Thermal correction to Enthalpy=                  0.263006
 Thermal correction to Gibbs Free Energy=         0.214254
 Sum of electronic and zero-point Energies=           -405.909339
 Sum of electronic and thermal Energies=              -405.897906
 Sum of electronic and thermal Enthalpies=            -405.896962
 Sum of electronic and thermal Free Energies=         -405.945714
```

TS_14

```
Zero-point correction=                           0.256992
(Hartree/Particle)
 Thermal correction to Energy=                    0.267829
 Thermal correction to Enthalpy=                  0.268773
 Thermal correction to Gibbs Free Energy=         0.221719
 Sum of electronic and zero-point Energies=           -405.949928
 Sum of electronic and thermal Energies=              -405.939091
 Sum of electronic and thermal Enthalpies=            -405.938146
 Sum of electronic and thermal Free Energies=         -405.985200
```
